# Suicide Behavior and Its Predictors in Patients with Schizophrenia in Ethiopia

**DOI:** 10.1155/2021/6662765

**Published:** 2021-04-01

**Authors:** Mohammed Ayalew, Semira Defar, Yared Reta

**Affiliations:** ^1^Department of Psychiatry Nursing, School of Nursing, College of Medicine and Health Sciences, Hawassa University, Hawassa, Ethiopia; ^2^Department of Midwifery, College of Health Sciences, Arsi University, Asella, Ethiopia

## Abstract

**Background:**

People with schizophrenia (PWS) are at greater risk of suicide. However, suicide behaviors that occur in PWS are often overlooked, inadequately characterized, and not consistently integrated into treatment. Despite this burden and consequences in Ethiopia, there is a dearth of studies concerning suicide behavior in PWS. Therefore, this study is aimed at assessing the magnitude of suicide behavior and its predictors among PWS in Ethiopia.

**Methods:**

An institution based cross-sectional study was employed. Data were collected using the structured interviewer-administered questionnaire from a sample of 300 PWS at Amanuel Mental Specialized Hospital (AMSH). The revised version of Suicide Behavior Questionnaire (SBQ-R) was used to assess suicide behavior in PWS. The data was collected from March 1 to 30, 2019. Binary logistic regression was performed to identify independent predictors of suicidal behavior at 95% confidence level. Statistical significance was declared at *p* value <0.05.

**Result:**

A total of 300 patients with schizophrenia participated in the study. More than two-thirds of 203 (67.7%) of the participants were males, and 116 (38.7%) participants were between the ages of 28 and 37 years. We found that the prevalence of suicide behavior among PWS was 30.3%. Being unemployed (AOR = 3.65, CI = 1.32, 10.05), family history of suicide (AOR = 3.16, CI = 1.38, 7.23), substance use (AOR = 2.51, CI = 1.13, 5.59), current positive psychotic symptoms (hallucination (AOR = 6.39, CI = 2.86, 14.29), and delusion (AOR = 4.15, CI = 1.95, 14.29) and presence of comorbid depression (AOR = 4.81, CI = 1.98, 11.68) were independent significant predictors with suicidal behavior in PWS.

**Conclusion:**

The prevalence of suicidal behavior among PWS was found to be high. Hence, designing strategies for early screening and intervention is the most critical prevention strategy of suicide in PWS.

## 1. Introduction

Suicide is an intension or act of deliberately ending one's own life, and suicide behavior is a feeling or intention that increases a person's risk of attempting or committing suicide, which may include suicidal thoughts or wishes, suicidal plans, and suicidal attempts [1, 2]. Suicide is a highly prevalent and significant public health problem resulting in nearly a million deaths per year [3]. Suicide is responsible for enormous distress to individuals, families, friends, and community [4].

Suicide behavior in patients with schizophrenia (PWS) is often due to the momentous psychosocial and economic burden on individuals and families [5]. Patients with schizophrenia die much earlier than expected [6], and up to 40% of these premature deaths can be due to suicide [7]. Studies estimated that nearly half (50%) of PWS attempt suicide [8], and around 10% completed suicide during their lifetime [9]. Epidemiological investigations indicated that PWS have about eight times increased risk of suicide than the general population [10] due to the complex and challenging nature of the symptoms of the disorder. During the course of the illness, 40% to 80% of PWS had suicidal ideation. Suicidal ideations are very common in first psychotic episode [10–13].

Suicide attempts occur in 20% to 50% of individuals having suicidal ideation, and 4% to 13% of PWS die from suicide [11]. In a retrospective medical record review of patients with schizophrenia, Yildiz et al. found out that about half (52%) and 28% had suicidal behavior (both suicidal thought or attempt) and attempted suicide at least once during the course of the disease, respectively [14]. Most suicide behaviors for PWS occur within the first ten years, and 50% happen in the first two years after onset of the illness [15, 16]. Also, the risk of committing suicide is high in the first weeks or months after discharge from the hospital [17]. In one follow-up study, 80% of PWS committed suicide while they were in hospital or in the first six months after discharge from hospital [18], and this may be as a result of “postpsychotic depression” [19, 20].

Previous studies in Ethiopia indicated that the rate of completing suicide was 7.7/100,000 in Addis Ababa from police and hospital records [21]. Suicide ideation and attempt for an urban community in Addis Ababa were indicated to be 2.7% and 0.9%, respectively [22], and suicide attempt was 3.2% for the adult population in rural Butajira [23]. Another study from Gondar psychiatric clinic found out that lifetime prevalence of suicidal ideation and attempted suicide was 64.8% and 19.2%, respectively [24]. In a study in Butajira among severe mental disorders, suicide attempt was found to be 26.3%, 23.8%, and 13.1% for major depression, bipolar-I disorders, and schizophrenia, respectively [25], while a study in south-west in people with mental illness showed 28.6% of suicide behavior [26].

Suicidal ideation is related with a greater risk of suicide attempts and can be considered as an early sign of suicidal behavior [27]. Even though the risk of suicide behavior in PWS is long-lasting, most patients completed suicide in the early course of the illness [28]. Moreover, PWS who attempt suicide seem to be more impulsive [29]. PWS were twice more likely to use a more violent method of attempting suicide (jumping, stabbing, hanging, firearm) [30].

Studies identified many predictors for suicide behavior in PWS such as affective disorders, recent loss, previous history of suicide attempt, substance use, feeling of nervousness or restlessness, and poor compliance for treatment [31]. Young age, being male, early age of onset illness, and multiple episodes of illness are commonly associated with suicide in PWS [8, 32]. Likewise, comorbid depression, being single, gradual onset of illness, social withdrawal, being hopeless, experiencing paranoid delusion, substance use, comorbid chronic disease, family history of suicide, and poor compliance to treatment are found to have a relationship with suicide [33]. PWS with prominent positive psychotic symptoms such as delusion of control, thought insertion, loosening of association, and flight of ideas had higher rate of completed suicide [34], and suicide attempt was associated with hostility rather than depression and negative symptoms [35, 36].

PWS are at a greater risk of suicide. Despite this burden and consequences in Ethiopia, there is a dearth of studies concerning suicide behavior in PWS. However, suicide behaviors that occur in PWS are often overlooked, inadequately characterized, and not consistently integrated into treatment. Therefore, recognizing suicide behavior in PWS may promote the early notification and intervention and thus prevent subsequent complications due to suicide. The most remarkable result of this study is that it alerts clinicians about suicidal behavior in PWS.

Even though Ethiopia developed national mental health strategy in 2012 to meet the mental health needs within Ethiopia [37], there is no organized/coordinated suicide prevention strategy developed. Severe mental disorders like schizophrenia may contribute many deaths by suicide. This study therefore gives a basic insight about suicide behavior in PWS, which helps clinicians to early identification and effectively manage suicide. Moreover, it helps policy makers, organizations working in the field, and future researchers as a baseline information and to develop effective national strategy in fighting against suicide. Therefore, the aim of this study was to assess the prevalence of suicide behavior and its predictors in PWS at Amanuel Mental Specialized Hospital (AMSH), Ethiopia.

## 2. Methods

### 2.1. Study Design, Area, and Period

A hospital-based cross-sectional study was employed from March 1 to 30, 2019, at AMSH in Addis Ababa, Ethiopia. The hospital is one of the eldest hospitals established in 1930 E. C., and it is the only mental specialized hospital in Ethiopia. On average, 46,520 patients with psychiatric disorders are visiting as an outpatient treatment yearly, and around 160 patients are admitted to the ward per month. The hospital has more than 300 beds that serve all types of mental disorders from all over the country. The hospital has 13 outpatient departments (OPDs). The hospital is also used as a teaching hospital and research center for mental health sciences.

### 2.2. Population

All PWS who have a follow-up visit at AMSH were the source population, and all sampled PWS who have a follow-up visit at AMSH during the study period and fulfill the inclusion criteria were considered as the study population.

### 2.3. Inclusion and Exclusion Criteria

PWS at OPD age 18 years and above were included. However, PWS, who were on severe acute psychotic episodes and who could not communicate, were excluded from the study. To assess the severity of the psychotic episode, the 1-item Clinical Global Impression Severity (CGI-S) scale with a seven-point liker scale question was used. CGI-S has a range of answers from 1 (normal) to 7 (amongst the most severely ill) [38]. Based on CGI-S, patients with a score of 6 or more were not included in the study.

### 2.4. Sample Size Determination and Sampling Technique

A total of 300 PWS were selected using a consecutive sampling technique from AMSH OPD. PWS who had follow-up visit at psychiatry OPD during the study period and who fulfilled the inclusion criteria were included in the study till the final sample size was reached.

### 2.5. Data Collection Tools and Quality Assurance

Data was collected using an interviewer-administered structured questionnaire. The questionnaire contains six parts: part one includes questions related to patients' sociodemographic characteristics and related factors, part two includes patient clinical characteristics, part three includes social support of the patient, part four medication includes adherence of the patients, part five includes items related to patients' suicide behavior, and part six includes questions to screen depression. The patient diagnosis was made using the Diagnostic Statistical Manual for Mental Disorders-5 (DSM-5) criteria by psychiatrists, psychiatry residents, and senior or expert mental health professionals.

The four items Suicidal Behavior Questionnaire*-*Revised (SBQ-R) was used to assess suicide behavior in PWS. SBQ-R item 1 assesses lifetime suicidal ideation and attempt, item 2 assesses the frequency of suicidal ideation over the past one year, item 3 describes the threat of suicide behavior, and item 4 evaluates the self-reported future probability of suicidal behavior. SBQ-R sensitivity and specificity were 80% and 91%, respectively, with a score ranges from 3 to 18. A cut-off score 8 or above is considered to determine the presence of suicide behavior for the adult clinical population [39].

Calgary Depression Scale for Schizophrenia (CDSS) was used to measure the level and intensity of depressive symptomatology in PWS. CDSS contains of 9 items that can be measured with a Likert-type scale (0 = absent, 1 = mild, 2 = moderate, and 3 = severe). The score ranges from 0 to 27 [40]. A score of 6 and above on the CDSS has been suggested to separate the comorbidity of depressive symptoms in PWS [41].

The level of social support among PWS was assessed using the three items Oslo Social Support Scale (OSSS), and the scores range from 3 to 14. A score of 3-8, 9-11, and 12-14 is considered as poor, moderate, and strong social support [42]. Determining medication adherence of PWS 4-items adapted from previous studies was used. The four items had a ^“^Yes^”^ = 0 and ^“^No^”^ = 1 scoring scheme. The 4-items are summed up to give a range of scores between 0 and 4, and scores ≤ 1 are considered as adhered, and score ≥ 2 is considered as nonadhered [43, 44].

The English version questionnaire was translated into the local language (Amharic) and back-translated to English by an independent translator to check for consistency and understandability of the questionnaire. The data were collected by psychiatry nurses and supervised by expert mental health specialists. Intensive training was given for the data collectors and supervisors before the actual data collection. A pretest was done at AMSH among PWS at the psychiatric ward before the actual data were collected to recognize impending problems and to check the consistency of the tools and the performance of the data collectors. Besides these, the data collectors were supervised on daily basis, and the filled questionnaires were checked by the supervisors and principal investigator daily.

### 2.6. Data Processing and Analysis

The collected data were checked, coded, and entered into Epi-data version 3.1 and exported to SPSS version 20 for cleaning and analysis. The patients' sociodemographic and clinical characteristics were analyzed using descriptive statistics, i.e., frequencies and percentages were calculated for the categorical variables. Independent bivariate logistic regression analysis was done for each independent variable against the dependent variable. In simple binary logistic regression analysis, variables with *p* value <0.05 were considered as a candidate for multiple logistic regressions to establish the variables that independently predict suicide behavior. The findings of the multivariate logistic regression were presented using the crude odd ratio (COR) and adjusted odds ratios (AOR). In multivariate logistic regression analysis, statistical significance was declared at 95% CI with *p* value <0.05. Finally, the results of the study were presented by tables, graphs, and narrative descriptions.

## 3. Results

### 3.1. Sociodemographic Characteristics of Participants

A total of 300 PWS participated in the study. More than two-thirds of 203 (67.7%) of the participants were males, and 116 (38.7%) participants were between the ages of 28 and 37 years. The majority 179 (59.7%) of the participants were single, 238 (79.3%) of the study participants were unemployed, and 244 (81.3%) were living with their family (Table 1).

### 3.2. Clinical Characteristic of Study Participants

Of all participants, 176 (58.6%) have >5-year duration of illness, about one-fourth 76 (25.3%) had a family history of mental illness, and 84 (28%) of participants use at least one type of substances in the past 12 months. Among the most common symptoms, one-third of 99 (33%) and 102 (34%) have hallucinations and anhedonia, respectively (Table 2).

### 3.3. Prevalence of Suicide Behavior in PWS

The overall lifetime suicidal ideation or attempt was 128 (42.8%). Furthermore, the lifetime suicidal attempt was 53 (17.7%), and the overall suicidal ideation over the past one year was 87 (29.0%) among PWS. The overall prevalence of suicide behavior in this study was found to be 30.3% in PWS (as shown in Table 3 below).

### 3.4. Independent Predictors of Suicide Behavior in PWS

Among variables that run in both bivariate and multivariate logistic regression analyses, being unemployed (AOR = 3.65, CI = 1.32, 10.05), family history of suicide (AOR = 3.16, CI = 1.38, 7.23), substance use (AOR = 2.51, CI = 1.13, 5.59), current positive psychotic symptoms (hallucination (AOR = 6.39, CI = 2.86, 14.29), and delusion (AOR = 4.15, CI = 1.95, 14.29)) and presence of comorbid depression (AOR = 4.81, CI = 1.98, 11.68) were the significant predictors of suicide behavior in PWS (Table 4).

## 4. Discussion

Suicidal behavior of PWS is a topic of tremendous significance because suicide is an irreversible condition, and should, for that reason, be prevented. Therefore, this study is aimed at determining the relationship between suicidal behavior and schizophrenia using representative institution-based data among Ethiopian PWS. The magnitude of suicidal behavior in PWS varies across different studies, based on the terminology used and the methodology of the study. Reports from WHO indicate that every 40 seconds, an individual died by suicide anywhere on the globe [45]. Likewise, deaths by suicide and suicide attempts highly affect families, communities, and societies negatively. It needs comprehensive strategy for the prevention of suicidal behavior in schizophrenia such as recognizing patients at high risk, delivering the best possible therapy, and family interventions. Also, it is important to educate mental health and nonmental health providers about suicide prevention strategies [46].

In this study, we found a high rate of suicide behavior, with nearly one-third (30.3%) of PWS. This finding was similar to a study among people with mental illness in Jimma (28.6%) in a similar setting [26]. Even though there is a potential underreporting of suicide behavior in most societies [26], suicide is a major public health challenge in PWS. The lifetime prevalence of suicidal ideation or attempt among PWS in this study was found to be (42.7%). This was almost comparable in a community representative study in Canada (39.2%) in a similar population [47].

Suicide is familiar in PWS, and it is projected that around 10% of PWS will, in the long run, commit suicide, and about fourfold of that figure makes an attempt [17]. Also, our finding of lifetime prevalence of suicide attempts was 17.7%. Similar estimates were reported in Jimma [26] and Gondar in people with mental illness [48]. Also, a similar study in Tukey found out 18.3% history of suicide attempts [49]. However, slightly lower suicidal attempts (13%) were reported in the Butajira study [25]. Likewise, we found out that nearly one-third (29%) of patients had suicidal ideation in the past year that was comparable with studies in Jimma [26] and Mettu [50]. This was similar with Turkish (31.6%) study in a similar population and setting [49]. This indicates that health care services require to integrate suicide prevention strategies as an essential element. Communities play a central role in suicide prevention by providing adequate social support to people with mental disorders such as schizophrenia and engage in follow-up care, fighting against stigma, and support those bereaved by suicide [45]. Consequently, the assessment and management of PWS who have suicide behavior are essential. So, developing comprehensive suicide prevention strategy is crucially important as recommended by the WHO [45].

Although the individual suicide behavior can usually not be anticipated, identification and recognition of predicting features for suicidal behavior in PWS are the main components of prediction and prevention of suicide [51]. Such efforts are especially significant in PWS for the reason that suicidal behaviors are not commonly communicated [52]. A number of studies were indicated that PWS, who committed suicide, were seen unsuspecting by clinician a number of days before the suicide [53]. In general, one can distinguish amongst suicide risk factors of clinical population and those typical to schizophrenia [54].

Similar to a previous study [55], in our study, we examined that suicide behavior was significantly more common in PWS who were unemployed. Being without a job may confer risk of suicide by increasing the impact of stressful life events [56]. Another study also showed that about twofold increases the risk of suicide among the unemployed [57].

Suicide behavior was more likely to happen in patients with a family history of suicide as compared with their counterparts. Our finding seemed to be similar to studies showing that suicide history in the family rises risk of suicidal behavior in PWS [58, 59]. Fabien T. and his colleagues in their study indicated that a family history of suicide was significantly increased the risk of a history of suicide attempt, higher lethality, and frequency of suicide attempts [60].

Substance use disorder is often the common comorbid problem in PWS and found to be associated with suicide behavior. PWS who had comorbid substance abuse were significantly more suicidal than their counterparts [33]. Similarly, this study indicated that PWS who use any type of substance was 2.5 times more likely to have suicide behavior. Nock and his colleagues in the WHO Mental health survey found out that substance abuse, such as alcohol and drug use, are substantially associated with suicide [61]. The greater risk of suicide behavior in substance user PWS [62, 63] may perhaps be the collective effect of several features, such as the loss of emotional control due to the use of psychoactive substances, nonadherence with psychotropic drugs, and existence of paranoid behavior and depressive symptoms [64]. Substance use in schizophrenia gets worse both positive psychotic symptoms, and the prognosis of the disease is linked with increasing relapse rates [33]. Moreover, PWS who use substances develop more psychotic symptoms, particularly hallucinations [65] and more frequent suicide attempts compared to their counterparts [65, 66].

In this study, positive psychotic symptoms, i.e., hallucinations and delusions were significantly associated with suicide behavior in PWS. Previous studies also found out that hallucinations and delusions are common in PWS who committed suicide [34, 67]. Suicide behavior is commonly precipitated by being distressed by positive symptoms such as command hallucinations [55]. The occurrence of psychotic symptoms, specifically auditory hallucinations and delusions in PWS, increases the risk of suicide behavior [68]. It has been understood for a long that positive psychotic symptoms are specific predictors for suicide in PWS [17]. Hence, a patient with command auditory hallucination such as a voice commanding him/her to kill himself/herself has been long recognized to be particularly held responsible for suicidal behavior in PWS [27]. Several studies demonstrated that the positive features in schizophrenia increase suicide risk, and 80% of PWS have delusions during suicidal attempts [31, 69–71].

Besides, depression is the major and most common risk factor for suicide behavior in PWS [68]. Also, depressive symptoms have been highly connected with suicide in PWS [72]. Hence, in our study, we found out that the cooccurrence of depression with schizophrenia 4.8 times increases the risk of suicide behavior. It is supposed that depression and loss of interest could result in demoralization, in turn, causes feelings of hopelessness and suicide [73]. Also, feeling of hopelessness and perceived distress was proposed to increase the risk of suicide in PWS [74]. Similarly, loss of confidence in the treatment and truthful awareness of the disease is often augmented by depression as distinct risk factors for suicide [54]. In one study, about one-fourth of patients who attempted suicide reported that they had attempted to commit suicide because of depression symptoms [49]. Despite the fact that suicide prevention in PWS is a complex task, clinicians need to be aware in how to recognize patients who are at increased risk of suicide behavior. Therefore, clinicians need to be vigilant in the management of psychotic symptoms, comorbid depression, and substance use disorders to prevent suicide in PWS [46].

Even though this study provided a baseline data, it also has some limitations encountered. Due to the retrospective nature of suicide attempts and early adversities, recall bias may have occurred. In addition, social desirability bias, for instance, the data were collected through interviewer-administered method that the participants might reply to either overreporting or underreporting.

## 5. Conclusions and Recommendations

A high prevalence of suicidal behavior among PWS was observed. We found that being unemployed and have a family history of suicide, comorbid depressive symptoms, active positive psychotic symptoms (hallucinations and delusions), and coexisting use of substance have the strongest association with suicide behavior in PWS. Early detection and intervention are the most critical prevention strategies of suicide in patients with schizophrenia. Thus, clinicians are strongly recommended in recognizing those patients with the high risk factors distinguished above and aggressively managing any comorbid depressive and positive psychotic symptoms, along with addressing any concurrent substance abuse. On the other hand, prediction of suicide in PWS is multifaceted, and efforts at prevention should as well give emphasis on optimizing employment opportunity of these individuals.

## Figures and Tables

**Table 1:** Sociodemographic characteristics of PWS at AMSH, 2019 (*n* = 300).

Variable	Categories	Frequency	Percentage (%)
Sex	Male	203	67.7
	Female	97	32.3

Age in years	18-27 years	61	20.3
	28-37 years	116	38.7
	38-47 years	82	27.3
	≥48 years	41	13.7

Marital status	Single	179	59.7
	Married	92	30.7
	Divorced	29	9.6

Educational status	Illiterate	70	23.3
	1-8^th^ grade	75	25.0
	9-12^th^ grade	122	40.7
	College and above	33	11.0

Occupation	Employed	62	20.7
	Unemployed	238	79.3

Place of residence	Rural	78	26
	Urban	222	74

Living status	With family	244	81.3
	Alone	56	18.7

**Table 2:** Clinical characteristics of patients with schizophrenia at AMSH, Addis Ababa, 2019 (*n* = 300).

Variable	Categories	Frequency	Percentage
Duration of illness	≤3 years	74	24.7
	3-5 years	50	16.7
	>5 years	176	58.6

Family history of mental illness	Yes	76	25.3
	No	224	74.7

Family history of suicide	Yes	67	22.3
	No	233	77.7

Episodes of illness	Continuous	74	24.7
	Single episode	82	27.3
	2-4 episodes	87	29
	5 and above	57	19

Any substance use in the past 12 months	Yes	84	28.0
	No	216	72.0

Medication adherence	Adhered	232	77.3
	Nonadhered	68	22.7

Social support	Poor	188	62.6
	Moderate	101	33.7
	Strong	11	3.7

Positive symptoms of schizophrenia			
Delusional symptoms	Yes	90	30
	No	210	70
Hallucinatory symptoms	Yes	99	33
	No	201	67
Disorganized speech or behavior	Yes	26	8.7
	No	273	91

Negative symptoms of schizophrenia			
Anhedonia	Yes	102	34
	No	197	65.7
Asocial	Yes	84	28
	No	216	72
Loose of personal motivation	Yes	67	22.3
	No	232	77.3
Loose of verbal expression	Yes	35	11.7
	No	264	88

Depression	Yes	92	30.7
	No	208	69.3

**Table 3:** Suicide behavior among patients with schizophrenia at AMSH, 2019 (*n* = 300).

S.no	Items	Category	Frequency	Percentage
1	Life time suicidal ideation, intent, and/or attempts	Never	172	57.3
		Suicidal ideation	51	17.0
		Suicide plan	24	8.0
		Suicide attempts	53	17.7
		Over all lifetime suicide ideation/attempt	128	42.6

2	Frequency of suicidal ideation in the past one year	Never	213	71.0
		Rarely (1 time)	53	17.7
		Sometimes (2 times)	25	8.3
		Often (3-4 times)	6	2.0
		Very often (5 or more times)	3	1.0
		One year all suicidal ideation	87	29.0

3	Threat of suicidal attempt	Never	276	92.0
		Once	18	6.0
		Two or more	6	2.0

4	Likelihood of suicide behavior in the future	Never	173	57.7
		No chance at all	14	4.7
		Rather unlikely	23	7.7
		Unlikely	16	5.3
		Likely	45	15.0
		Rather likely	22	7.3
		Very likely	7	2.3

Overall prevalence of suicide behavior		Yes	91	30.3
		No	209	69.7

**Table 4:** Bivariate and multivariate analyses for determinant factors of suicide behavior in patients with schizophrenia at AMSH, Addis Ababa, Ethiopia, 2019 (*N* = 300).

Variables	Category	Suicide behavior		COR (95% CI)	AOR (95% CI)
		Yes	No		
Age	18-27	16	45	1	1
	28-37	36	80	1.26 (0.63, 2.53)	1.44 (0.48, 4.32)
	38-47	24	58	1.16 (0.55, 2.45)	1.20 (0.34, 4.17)
	≥48	16	25	1.80 (0.77, 4.20)	1.29 (0.29, 5.73)

Sex	Male	61	142	1	1
	Female	31	66	1.09 (0.65, 1.84)	2.05 (0.29, 4.62)

Marital status	Single	47	132	1	1
	Married	32	60	1.50 (0.87, 2.58)	1.56 (0.67, 3.60)
	Divorced	13	16	2.28 (1.02, 5.09)	2.49 (0.73, 8.55)

Educational status	Illiterate	25	45	1.27 (0.52, 3.12)	1.07 (0.26, 4.41)
	Primary	25	50	1.15 (0.47, 2.78)	1.22 (0.34, 4.35)
	Secondary	32	90	0.82 (0.35, 1.90)	1.07 (0.31, 3.66)
	≥college	10	23	1	1

Occupational status	Employed	15	47	1	1
	Unemployed	77	161	1.49 (0.78, 2.85)	3.65 (1.32, 10.05)^∗∗∗^

Area of residence	Rural	28	50	1	1
	Urban	64	158	0.72 (0.42, 1.25)	0.85 (0.34, 2.07)

Living status	With family	75	182	1	1
	Alone	17	26	1.58 (0.81, 3.09)	1.78 (0.65, 4.93)

Duration of illness	≤3 years	20	54	1	1
	3.1-5 years	12	38	0.85 (0.37, 1.95)	1.99 (0.59, 6.67)
	>5 years	60	116	1.39 (0.76, 2.55)	2.49 (0.88, 7.03)

Known chronic illness	Yes	20	19	2.76 (1.39, 5.47)	1.37 (0.41, 4.56)
	No	72	189	1	1

Family history of mental illness	Yes	26	51	1.21 (0.69, 2.12)	1.05 (0.47, 2.35)
	No	66	157	1	1

Family history of suicide	Yes	47	5	3.67 (2.08, 6.48)	3.16 (1.38, 7.23)^∗∗^
	No	45	203	1	1

Episodes of illness	Continuous	19	56	1	1
	Single episode	29	53	1.70 (0.85, 3.42)	1.77 (0.66, 4.75)
	2-4 episodes	24	63	1.18 (0.58, 2.41)	0.52 (0.17, 1.58)
	5 and above	21	36	1.82 (0.85, 3.86)	1.29 (0.42, 3.98)

Substance use	Yes	42	46	2.96 (1.75, 5.00)	2.51 (1.13, 5.59)^∗∗∗^
	No	50	162	1	1

Social support	Poor	60	128	0.82 (0.23, 2.91)	0.40 (0.06, 2.66)
	Moderate	28	73	0.67 (0.18, 2.47)	0.35 (0.05, 2.44)
	Strong	4	7	1	1

Medication adherence	Adhered	65	159	1	1
	Nonadhered	27	49	1.35 (0.77, 2.34)	2.01 (0.86, 4.70)

Hallucination	Yes	62	48	6.89 (4.00, 11.85)	6.39 (2.86, 14.29)^∗^
	No	30	160	1	1

Delusion	Yes	56	39	6.74 (3.91, 11.62)	4.15 (1.95, 14.29)^∗^
	No	36	169	1	1

Disorganized speech	Yes	16	11	3.77 (1.67, 8.49)	1.72 (0.42, 7.03)
	No	76	197	1	1

Anhedonia	Yes	36	67	1.35 (0.81, 2.25)	0.44 (0.18, 1.03)
	No	56	141	1	1

Asocialia	Yes	31	53	1.48 (0.87, 2.53)	0.93 (0.37, 2.36)
	No	61	155	1	1

Loss of personal motivation	Yes	27	41	1.69 (0.96, 2.97)	0.53 (0.20, 1.38)
	No	65	167	1	1

Alogia	Yes	18	18	2.56 (1.26, 5.20)	1.96 (0.64, 6.04)
	No	74	190	1	1

Depression	Yes	45	163	6.08 (3.53, 10.46)	4.81 (1.98, 11.68)^∗^
	No	47	45	1	1

^∗^ *p* < 0.001, ^∗∗^*p* < 0.01, ^∗∗∗^*p* < 0.05.

## Data Availability

All the datasets used and analyzed during the current study are available on this manuscript.

## Abbreviations

AMSH: Amanuel Mental Specialized Hospital
AOR: Adjusted odd ratio
APA: American Psychiatry Association
CDSS: Calgary Depression Scale for Schizophrenia
CI: Confidence interval
COR: Crude odd ratio
DSM (5): Diagnostic and Statistical Manual of 5^th^ Edition
IRB: Institutional review board
OPD: Outpatient department
OR: Odd ratio
OSSS: Oslo Social Support Scale
SBQ-R: Suicidal Behavior Questioner Revised
SPSS: Statistical Package for Social Science
WHO: World Health Organization.
